# Complete Genome Sequence of the *p*-Nitrophenol-Degrading Bacterium *Pseudomonas putida* DLL-E4

**DOI:** 10.1128/genomeA.00596-14

**Published:** 2014-06-19

**Authors:** Xiaojun Hu, Jue Wang, Fei Wang, Qiongzhen Chen, Yan Huang, Zhongli Cui

**Affiliations:** a Key Laboratory of Microbiological Engineering of Agricultural Environment, Ministry of Agriculture, College of Life Sciences, Nanjing Agricultural University, Nanjing, People’s Republic of China; b College of Bioscience and Bioengineering, Jiangxi Agricultural University, Nanchang, People’s Republic of China

## Abstract

The first complete genome sequence of a *p*-nitrophenol (PNP)-degrading bacterium is reported here. *Pseudomonas putida* DLL-E4, a Gram-negative bacterium isolated from methyl-parathion-polluted soil, can utilize PNP as the sole carbon and nitrogen source. *P. putida* DLL-E4 has a 6,484,062 bp circular chromosome that contains 5,894 genes, with a G+C content of 62.46%.

## GENOME ANNOUNCEMENT

*Pseudomonas putida* is one of the best-studied species of the metabolically versatile and ubiquitous genus of *Pseudomonas*. *p*-Nitrophenol (PNP) is the most common and important pollutant in terms of the quantities manufactured and the extent of environmental contamination. It is widely used in the synthesis of medicines, dyes, explosives, leather coloring, wood preservatives, and rubber chemicals (1). Various bacteria, including *Burkholderia* spp., *Rhodococcus opacus*, and *Pseudomonas* spp., have been studied for their PNP-degrading capacities (2–6), and the genetic potential of *P. putida* DLL-E4 can be exploited for the biodegradation of PNP and related pollutants. To this end, the whole-genome sequence will aid in characterizing the genomic and metabolic diversity of this species.

The genome of *P. putida* DLL-E4 was sequenced by using a hybrid strategy with Roche 454 GS FLX Titanium (total reads, 41,221,198; 8-kb paired-end reads) and Illumina HiSeq 2000 (total reads, 18,946,756; 150-bp paired-end reads) platforms. The 454 sequence data were assembled with the Newbler assembler, whereas the Solexa data were assembled to contigs with the Velvet program. In all, 258 contigs were generated through the *de novo* assembly. Gaps between the contigs were closed by primer walking on standard PCR products. The open reading frames were predicted with Glimmer 3.02 (7) and annotated through comparisons to the NCBI-NR database (8). The metabolic pathways of aromatic and heterocyclic compounds were analyzed using the Kyoto Encyclopedia of Genes and Genomes database (9).

The problem of large gaps predicted by MUMmer and BLAST was solved by using ContigScape (10), which interactively displays the relationships between genomic contigs, thereby allowing a faster and more precise determination of linkages and greatly improving the efficiency of gap closing (10). Gap closure was verified by PCR amplification.

The genome of strain DLL-E4 consists of a single circular chromosome that is 6,484,062 bp in length and has a G+C content of 62.46%, containing 5,979 genes, which includes 5,894 coding sequences, 69 tRNA genes, and 5 rRNA operons. Among the 4,823 genes with predicted functions were those related to the catabolism of aromatic and heterocyclic compounds, such as PNP and hydroquinone (HQ). Two PNP degradation gene clusters, *pnp* and *pnp1*, were found between bp 3906279 and 3943639  in the genome. PNP can be aerobically degraded by the 1,2,4-benzenetriol (11, 12) and HQ (13) pathways. The *pnp* and *pnp1* gene clusters contain a combination of genes from both pathways (6).

Fourteen genomic islands (GIs) were identified in the genome: the largest (24,188 bp in length) has a G+C content of 58% and contains genes for four transposases. *pnp1* is located in this GI (our unpublished data), and the *pnp* gene clusters were found between this and another GI. This finding suggests that the *pnp* gene clusters were originally located in the chromosome, whereas the *pnp1* gene clusters were exogenous DNA integrated into the genome.

Further analyses of these genes will provide insight into the mechanism of PNP degradation.

### Nucleotide sequence accession number.

The complete genome sequence of *P. putida* DLL-E4 has been deposited in the GenBank database under the accession no. CP007620.
